# Severe Post-cardioversion Bradycardia: A Case Report Highlighting the Anesthesiology Perspective

**DOI:** 10.7759/cureus.92478

**Published:** 2025-09-16

**Authors:** Ali S Jafri, Justin Tai, Abdul-Haseeb Sheikh

**Affiliations:** 1 Anesthesiology, Touro College of Osteopathic Medicine, Middletown, USA; 2 Anesthesiology, Vassar Brothers Medical Center, Poughkeepsie, USA

**Keywords:** atrial fibrillation, bradycardia, cardioversion, isoproterenol, sick sinus syndrome

## Abstract

Electrical cardioversion is commonly used to restore sinus rhythm in atrial fibrillation but can rarely precipitate clinically significant bradycardia. We present the case of an 83-year-old female with atrial fibrillation, severe mitral regurgitation, heart failure with preserved ejection fraction (HFpEF), hypertension, gout, and hypothyroidism who was recently admitted with acute decompensated heart failure. She underwent synchronized cardioversion under procedural sedation with propofol and ketamine. Immediately after conversion, she developed symptomatic bradycardia with heart rates in the 30s and hypotension. Multiple doses of atropine and vasopressor support provided only transient improvement. Temporary pacing was attempted but proved insufficient, and an isoproterenol infusion was initiated, stabilizing both heart rate and blood pressure. She was admitted to the ICU for monitoring and subsequently underwent permanent pacemaker implantation.

This case underscores the importance of recognizing conduction abnormalities in the peri-cardioversion period, particularly in elderly patients with structural heart disease and those receiving atrioventricular (AV) nodal blocking agents. It highlights the anesthesiology perspective on acute management, emphasizing a stepwise pharmacologic approach, preparedness for pacing, and the role of isoproterenol infusion in refractory cases. Prompt recognition and escalation of therapy are essential to prevent hemodynamic compromise and improve patient outcomes.

## Introduction

Atrial fibrillation is the most common sustained arrhythmia encountered in clinical practice and is associated with increased morbidity and mortality [1]. Cardioversion, either pharmacologic or electrical, is a widely used strategy to restore sinus rhythm [2]. However, bradyarrhythmias may complicate the procedure, manifesting as sinus arrest, high-grade atrioventricular (AV) block, or sick sinus syndrome [3]. Patients with structural heart disease or preexisting conduction abnormalities are particularly vulnerable [4].

Proposed mechanisms of bradyarrhythmia following cardioversion include unmasking of latent sinus node dysfunction, increased vagal tone, abrupt withdrawal of atrial pacing, and underlying conduction disease in the setting of valvular pathology. While cardiology teams typically determine long-term rhythm management, anesthesiologists are primarily responsible for immediate hemodynamic stabilization during and after the procedure. This case highlights the anesthesiology perspective in the acute management of severe post-cardioversion bradycardia, underscoring the role of isoproterenol infusion as a rescue therapy.

## Case presentation

An 83-year-old female with a history of persistent atrial fibrillation/flutter on rivaroxaban, HFpEF (EF >52%) with severe mitral regurgitation, severe right ventricular dysfunction, moderate pulmonary hypertension, hypertension, hyperlipidemia, gout, and hypothyroidism presented for transesophageal echocardiogram (TEE) and elective cardioversion. She had recently been hospitalized for acute decompensated heart failure requiring IV diuresis.

Medications

Home Medications

Metoprolol tartrate 50 mg BID, rivaroxaban 20 mg daily, olmesartan 20 mg daily, levothyroxine 25 mcg daily, febuxostat 40 mg daily, fenofibrate 54 mg daily, oxybutynin ER 10 mg daily.

Inpatient Medications

losartan 50 mg daily, IV furosemide 40 mg BID, metoprolol succinate 50 mg BID, enoxaparin 50 mg BID. She was not taking digoxin, calcium channel blockers, or class I/III antiarrhythmics.

Sedation and procedure

Standard sedation for cardioversion was achieved with propofol 50 mg and ketamine 20 mg, maintaining spontaneous ventilation and airway patency with continuous monitoring per guidelines. One unit of vasopressin was administered for blood pressure support. The patient tolerated the TEE portion with stable vitals (~120s/90s).

**Figure 1:**
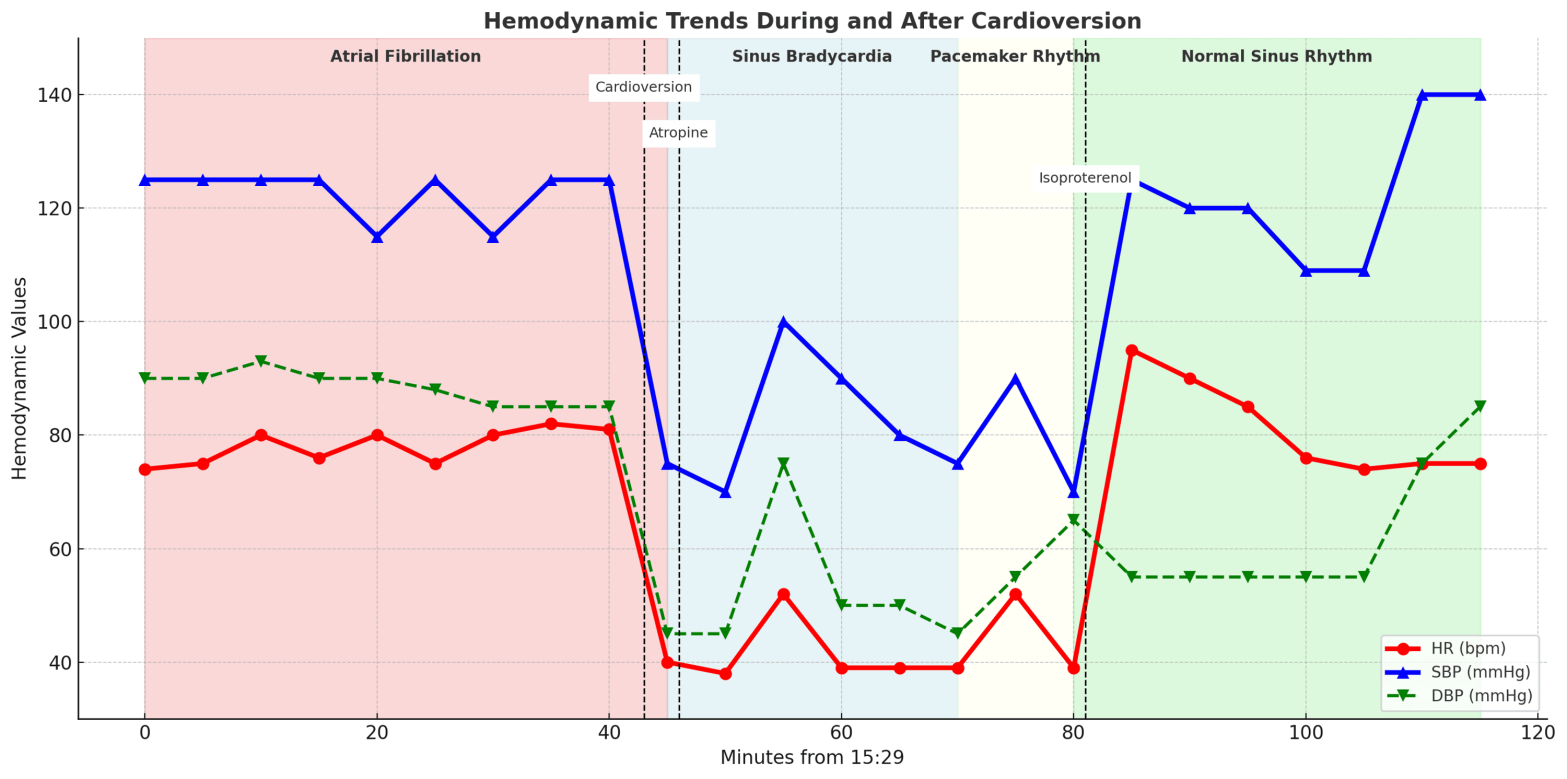
Hemodynamic trends and rhythm transitions during and after cardioversion. Heart rate (red) and blood pressure (systolic: blue, diastolic: green) are plotted against time. Cardioversion at 16:11 converted atrial fibrillation to sinus bradycardia, followed by administration of atropine and vasopressin at 16:15. Hemodynamics stabilized after initiation of isoproterenol at 16:50, with recovery to normal sinus rhythm. Rhythm transitions are indicated by shaded overlays: atrial fibrillation (red), sinus bradycardia (blue), paced rhythm (white), and normal sinus rhythm (green). Norepinephrine (16 µg) and epinephrine (100 µg) boluses were administered before isoproterenol but are not shown on the chart for clarity; their limited effect is described in the Case Presentation.

At 16:11, synchronized cardioversion was performed, converting atrial fibrillation to sinus bradycardia (HR ~40 bpm, SBP ~75 mmHg). At 16:15, the first dose of atropine 1 mg was administered, producing only transient improvement. Despite repeated atropine dosing (five 1 mg boluses every 5-10 minutes, total 5 mg), hypotension persisted. Norepinephrine boluses (16 µg) and epinephrine boluses (100 µg) were administered with minimal effect. Transcutaneous pacing was applied with partial improvement. At 16:50, continuous isoproterenol infusion was initiated at 5 µg/min on cardiology’s recommendation, resulting in HR stabilization in the 90s with improved blood pressure, allowing discontinuation of pacing (Figure 1).

Outcome

The patient was admitted to the ICU for monitoring. Isoproterenol was gradually weaned off, and she remained in sinus rhythm with intermittent pauses. Electrophysiology later proceeded with dual-chamber permanent pacemaker implantation. She was discharged home in stable condition with outpatient cardiology follow-up.

## Discussion

Bradyarrhythmias are a recognized complication of cardioversion, particularly in patients with atrial enlargement, valvular disease, or other structural heart abnormalities [1,4]. In this patient, severe mitral regurgitation and a prior heart failure hospitalization may have predisposed her to conduction instability. Electrical cardioversion can unmask latent sinus node dysfunction, including sick sinus syndrome, which may not become apparent until atrial pacing is abruptly withdrawn [2,3]. Recognizing this mechanism is critical, as persistent bradyarrhythmia may necessitate permanent pacemaker implantation [3].

Although post-cardioversion bradycardia is often transient, profound episodes can result in hypotension and hypoperfusion, requiring prompt pharmacologic intervention [2,3]. Atropine is first-line therapy for symptomatic bradycardia but may only provide transient benefit. The AHA bradycardia algorithm recommends temporary pacing when instability persists; in this case, pacing was attempted but did not provide sustained stability. Escalation to vasoactive drugs was necessary. Norepinephrine and epinephrine boluses were administered but produced little effect, highlighting the limitations of vasopressor boluses for augmenting chronotropy. In contrast, isoproterenol provided selective beta-adrenergic stimulation, enhancing sinus node automaticity and AV conduction without imposing additional afterload, an advantage in a patient with severe mitral regurgitation. Its use stabilized hemodynamics until definitive pacemaker implantation, underscoring its utility as a temporizing therapy [3,4].

Observational data suggest that post-cardioversion bradycardia occurs in a small but clinically significant proportion of patients, with higher incidence in older adults and those with structural heart disease [1,2,4]. From an anesthesiology perspective, these events highlight the importance of rapid assessment of airway and perfusion, readiness for pharmacologic escalation, and preparation for pacing if needed [4]. A structured, stepwise approach during the peri-procedural period facilitates restoration of perfusion and hemodynamic stability. This case emphasizes the anesthesiologist’s role in acute bedside management, demonstrating the effectiveness of isoproterenol in refractory post-cardioversion bradycardia and underscoring the need for vigilance in patients with underlying conduction disease.

## Conclusions

This case illustrates the potential for profound bradycardia immediately following cardioversion in a high-risk patient with structural heart disease. From an anesthesiology perspective, prompt recognition, airway protection, hemodynamic stabilization, and stepwise escalation are essential. This case highlights the value of isoproterenol infusion as a stabilizing therapy when conventional measures, including atropine, norepinephrine, epinephrine, and temporary pacing, are insufficient.

## References

[1] Grönberg T, Nuotio I, Nikkinen M, Ylitalo A, Vasankari T, Hartikainen JE, Airaksinen KE (2013). Arrhythmic complications after electrical cardioversion of acute atrial fibrillation: the FinCV study. Europace.

[2] Poçi D, Abrahamsson BM, Edvardsson N, Bergfeldt L (2013). Sinus bradycardia and sinus pauses immediately after electrical cardioversion of persistent atrial fibrillation--what do they mean?. Ann Noninvasive Electrocardiol.

[3] Kusumoto FM, Schoenfeld MH, Barrett C (2019). 2018 ACC/AHA/HRS Guideline on the evaluation and management of patients with bradycardia and cardiac conduction delay: a report of the American College of Cardiology/American Heart Association Task Force on Clinical Practice Guidelines and the Heart Rhythm Society. Circulation.

[4] Panchal AR, Bartos JA, Cabañas JG (2020). Part 3: adult basic and advanced life support: 2020 American Heart Association guidelines for cardiopulmonary resuscitation and emergency cardiovascular care. Circulation.

